# Supplementary material for: Modeling glioblastoma heterogeneity as a dynamic network of cell states
Source: Mol Syst Biol. 2021 Sep 16;17(9):e10105. doi: 10.15252/msb.202010105 (PMC8444284; doi:10.15252/msb.202010105)
Supplement: Supplementary file 6 — Source Data for Figure 5 [file MSB-17-e10105-s004.zip › Figure5A_sourcedata/GSEA_3071/nosigdb_stateVI.GseaPreranked.1622795169056/RICHARDS_NOWAKOWSKI_SCIENCE_2017_MGE_IPC3_UPREG.html]

Details for gene set RICHARDS\_NOWAKOWSKI\_SCIENCE\_2017\_MGE\_IPC3\_UPREG[GSEA]

|  || Dataset | state63071 |
| Phenotype | NoPhenotypeAvailable |
| Upregulated in class | na\_neg |
| GeneSet | RICHARDS\_NOWAKOWSKI\_SCIENCE\_2017\_MGE\_IPC3\_UPREG |
| Enrichment Score (ES) | -0.5789465 |
| Normalized Enrichment Score (NES) | -2.758516 |
| Nominal p-value | 0.0 |
| FDR q-value | 0.0 |
| FWER p-Value | 0.0 |
Table: GSEA Results Summary

  

Fig 1: Enrichment plot: RICHARDS\_NOWAKOWSKI\_SCIENCE\_2017\_MGE\_IPC3\_UPREG      
 Profile of the Running ES Score & Positions of GeneSet Members on the Rank Ordered List

  

| PROBE | GENE SYMBOL | GENE\_TITLE | RANK IN GENE LIST | RANK METRIC SCORE | RUNNING ES | CORE ENRICHMENT || 1 | CITED2 |  |  | 5 | 1.232 | 0.0208 | No |
| 2 | RND3 |  |  | 49 | 0.866 | 0.0243 | No |
| 3 | HSPA5 |  |  | 414 | 0.484 | -0.0692 | No |
| 4 | PPCS |  |  | 450 | 0.471 | -0.0705 | No |
| 5 | ACTG1 |  |  | 508 | 0.453 | -0.0784 | No |
| 6 | CHD3 |  |  | 679 | 0.405 | -0.1188 | No |
| 7 | RPL4 |  |  | 746 | 0.387 | -0.1304 | No |
| 8 | CORO1C |  |  | 873 | 0.365 | -0.1592 | No |
| 9 | UPF3B |  |  | 998 | 0.346 | -0.1878 | No |
| 10 | CCND2 |  |  | 1064 | 0.335 | -0.2000 | No |
| 11 | PSMB2 |  |  | 1171 | 0.320 | -0.2240 | No |
| 12 | ACTB |  |  | 1243 | 0.310 | -0.2383 | No |
| 13 | NOP56 |  |  | 1531 | 0.276 | -0.3139 | No |
| 14 | PSMC2 |  |  | 1646 | 0.264 | -0.3411 | No |
| 15 | EIF1AX |  |  | 1657 | 0.263 | -0.3392 | No |
| 16 | RPL21 |  |  | 1725 | 0.258 | -0.3534 | No |
| 17 | HMGN2 |  |  | 1857 | -0.258 | -0.3855 | No |
| 18 | MOB3B |  |  | 1883 | -0.261 | -0.3878 | No |
| 19 | PDCL |  |  | 1915 | -0.266 | -0.3917 | No |
| 20 | SREBF2 |  |  | 2032 | -0.283 | -0.4192 | No |
| 21 | RNASEH2B |  |  | 2410 | -0.355 | -0.5186 | No |
| 22 | ABAT |  |  | 2438 | -0.365 | -0.5196 | No |
| 23 | NUCB2 |  |  | 2511 | -0.381 | -0.5330 | No |
| 24 | GLCCI1 |  |  | 2521 | -0.383 | -0.5286 | No |
| 25 | PBX3 |  |  | 2589 | -0.398 | -0.5403 | No |
| 26 | OXR1 |  |  | 2642 | -0.410 | -0.5475 | No |
| 27 | STMN1 |  |  | 2646 | -0.411 | -0.5410 | No |
| 28 | HAT1 |  |  | 2750 | -0.441 | -0.5619 | No |
| 29 | DLEU2 |  |  | 2804 | -0.459 | -0.5686 | No |
| 30 | CENPF |  |  | 2842 | -0.472 | -0.5705 | Yes |
| 31 | CASD1 |  |  | 2857 | -0.479 | -0.5658 | Yes |
| 32 | TPR |  |  | 2900 | -0.494 | -0.5687 | Yes |
| 33 | ILDR2 |  |  | 2904 | -0.496 | -0.5606 | Yes |
| 34 | BEST3 |  |  | 2911 | -0.498 | -0.5533 | Yes |
| 35 | ARX |  |  | 2960 | -0.520 | -0.5574 | Yes |
| 36 | MSI2 |  |  | 2992 | -0.537 | -0.5565 | Yes |
| 37 | KLHL13 |  |  | 3000 | -0.542 | -0.5487 | Yes |
| 38 | NES |  |  | 3001 | -0.542 | -0.5390 | Yes |
| 39 | TMPO |  |  | 3022 | -0.552 | -0.5347 | Yes |
| 40 | RBL1 |  |  | 3063 | -0.569 | -0.5357 | Yes |
| 41 | RFTN2 |  |  | 3065 | -0.570 | -0.5257 | Yes |
| 42 | SOX4 |  |  | 3066 | -0.571 | -0.5154 | Yes |
| 43 | SH3BGRL |  |  | 3095 | -0.582 | -0.5128 | Yes |
| 44 | GINS2 |  |  | 3119 | -0.598 | -0.5085 | Yes |
| 45 | NRXN3 |  |  | 3127 | -0.603 | -0.4996 | Yes |
| 46 | PFN2 |  |  | 3215 | -0.685 | -0.5117 | Yes |
| 47 | CDCA7 |  |  | 3225 | -0.693 | -0.5018 | Yes |
| 48 | USP1 |  |  | 3233 | -0.699 | -0.4912 | Yes |
| 49 | CDK6 |  |  | 3234 | -0.704 | -0.4786 | Yes |
| 50 | CERS6 |  |  | 3258 | -0.718 | -0.4721 | Yes |
| 51 | H1FX |  |  | 3261 | -0.721 | -0.4597 | Yes |
| 52 | DLX6 |  |  | 3289 | -0.749 | -0.4538 | Yes |
| 53 | BAZ2B |  |  | 3294 | -0.751 | -0.4414 | Yes |
| 54 | RGS16 |  |  | 3304 | -0.765 | -0.4302 | Yes |
| 55 | CCNE2 |  |  | 3305 | -0.767 | -0.4164 | Yes |
| 56 | SOX2 |  |  | 3314 | -0.778 | -0.4047 | Yes |
| 57 | FBXW7 |  |  | 3315 | -0.779 | -0.3907 | Yes |
| 58 | H3F3A |  |  | 3342 | -0.807 | -0.3834 | Yes |
| 59 | HELLS |  |  | 3361 | -0.826 | -0.3736 | Yes |
| 60 | HES6 |  |  | 3384 | -0.864 | -0.3643 | Yes |
| 61 | SP9 |  |  | 3411 | -0.914 | -0.3551 | Yes |
| 62 | HMGB2 |  |  | 3414 | -0.922 | -0.3391 | Yes |
| 63 | CCDC88A |  |  | 3421 | -0.944 | -0.3238 | Yes |
| 64 | CHD7 |  |  | 3454 | -1.017 | -0.3145 | Yes |
| 65 | DLL3 |  |  | 3460 | -1.030 | -0.2974 | Yes |
| 66 | RAB3IP |  |  | 3493 | -1.094 | -0.2867 | Yes |
| 67 | CLSPN |  |  | 3499 | -1.115 | -0.2681 | Yes |
| 68 | SOX6 |  |  | 3517 | -1.174 | -0.2517 | Yes |
| 69 | DCLK2 |  |  | 3524 | -1.206 | -0.2317 | Yes |
| 70 | CKB |  |  | 3579 | -1.575 | -0.2186 | Yes |
| 71 | DLX1 |  |  | 3593 | -1.696 | -0.1917 | Yes |
| 72 | DLX2 |  |  | 3595 | -1.720 | -0.1611 | Yes |
| 73 | ASCL1 |  |  | 3604 | -1.822 | -0.1306 | Yes |
| 74 | INSM1 |  |  | 3607 | -1.872 | -0.0974 | Yes |
| 75 | DLX5 |  |  | 3623 | -2.501 | -0.0567 | Yes |
| 76 | LMO1 |  |  | 3635 | -3.371 | 0.0008 | Yes |
Table: GSEA details [plain text format]

  

Fig 2: RICHARDS\_NOWAKOWSKI\_SCIENCE\_2017\_MGE\_IPC3\_UPREG: Random ES distribution      
 Gene set null distribution of ES for **RICHARDS\_NOWAKOWSKI\_SCIENCE\_2017\_MGE\_IPC3\_UPREG**
